# Cure of Strabismus

**Published:** 1840-10

**Authors:** 


*Cure of Strabismus.*—M. Jules Guerin announces, in a letter recently addressed to the Academy of Sciences, that he has performed Dieffenbach's operation for the cure of strabismus in four cases with success.

I had long ago established, says M. Guerin, and publicly professed, that strabismus depends on retraction of the muscles of the eye, and its various forms depend on the different degrees of retraction, variously affecting the different muscles which move the eyeball. This is a simple application of my theory of the deformities of the joints in general, which elicited from a distinguished member of the Academy, the quaint, but just remark, that squinting was the club-foot of the eye. In accordance with this theory, I have proposed to extend to the eye the section of its muscles; the mode of operating which I have adopted, differs slightly from that of Dieffenbach; the results have been advantageous, but not immediately so: in one case only did the eye become quite straight soon after the operation; in others there was merely an amelioration, and this circumstance appears to me to be a natural consequence of the true origin of squinting.—*Journal des Debats. Ibid.*

